# Specific BK Channel Activator NS11021 Protects Rat Renal Proximal Tubular Cells from Cold Storage—Induced Mitochondrial Injury In Vitro

**DOI:** 10.3390/biom9120825

**Published:** 2019-12-04

**Authors:** Stephen Shrum, Nancy J. Rusch, Lee Ann MacMillan-Crow

**Affiliations:** Department of Pharmacology and Toxicology, College of Medicine, University of Arkansas for Medical Sciences, 4301 W. Markham Street, Mail Slot 611, Little Rock, AR 72205, USA; sashrum@uams.edu (S.S.); NRusch@uams.edu (N.J.R.)

**Keywords:** mitochondria, transplantation, kidney, cold storage, oxidative stress, respiration, mitoBK channel, NS11021

## Abstract

Kidneys from deceased donors used for transplantation are placed in cold storage (CS) solution during the search for a matched recipient. However, CS causes mitochondrial injury, which may exacerbate renal graft dysfunction. Here, we explored whether adding NS11021, an activator of the mitochondrial big-conductance calcium-activated K^+^ (mitoBK) channel, to CS solution can mitigate CS-induced mitochondrial injury. We used normal rat kidney proximal tubular epithelial (NRK) cells as an in vitro model of renal cold storage (18 h) and rewarming (2 h) (CS + RW). Western blots detected the pore-forming α subunit of the BK channel in mitochondrial fractions from NRK cells. The fluorescent K^+^-binding probe, PBFI-AM, revealed that isolated mitochondria from NRK cells exhibited mitoBK-mediated K^+^ uptake, which was impaired ~70% in NRK cells subjected to CS + RW compared to control NRK cells maintained at 37 °C. Importantly, the addition of 1 μM NS11021 to CS solution prevented CS + RW-induced impairment of mitoBK-mediated K^+^ uptake. The NS11021–treated NRK cells also exhibited less cell death and mitochondrial injury after CS + RW, including mitigated mitochondrial respiratory dysfunction, depolarization, and superoxide production. In summary, these new data show for the first time that mitoBK channels may represent a therapeutic target to prevent renal CS-induced injury.

## 1. Introduction

Seventy percent of kidneys used for renal transplantation are provided by deceased donors and are routinely subjected to cold storage (CS) preservation prior to transplantation (2018 UNOS website data). Cold storage organ preservation was made a clinical reality by the pioneering work of Belzer and Southard, which revolutionized the possibilities of organ transplantation [1,2,3,4]. The CS solution is designed to slow the metabolic rate of renal cells to protect donor kidneys from ischemic injury, thereby extending the window of time available to find a matched recipient (for a review, see Salahudeen et al. [5]). Indeed, the use of CS has greatly increased the number of available donor kidneys, and CS is considered a clinical necessity. However, CS causes renal injury, worsens renal dysfunction, and ultimately increases the chance of transplant failure 5-fold compared to transplanted kidneys that have not undergone CS [5,6,7]. Despite this evident problem, there is a lack of clinical interventions to protect donor kidneys from CS-induced damage. Thus, it is imperative to identify novel therapeutic targets to prevent CS-induced injury during renal transplantation.

In this regard, our laboratory and others have shown that mitochondrial injury is a central triggering event of CS-induced renal injury [8,9,10,11,12]. Using renal tubular cell lines exposed to CS followed by rewarming (CS + RW), and employing animal models of renal CS followed by transplantation (CS + Tx), numerous studies have indicated that CS induces mitochondrial reactive oxygen species (ROS), respiratory complex dysfunction, mitochondrial depolarization, Ca^2+^ overload, and cell death [8,9,10,11,12,13,14,15,16]. Since mitochondrial health is recognized as a feature essential for optimal viability of biological systems, interventions designed to protect renal mitochondria during CS are worth investigating as potential therapeutic approaches. For example, our earlier studies revealed that overexpression of the mitochondrial antioxidant, manganese superoxide dismutase (MnSOD), in renal tubular cells confers protection from CS-induced renal injury [10]. Similarly, the addition of the mitochondrial-targeted antioxidant, mito-ubiquinone (MitoQ), to the CS solution is renoprotective in vitro and ex vivo [14,15]. These findings and others highlight a contribution of mitochondrial ROS to CS-induced renal injury and also validate mitochondrial ROS as a valuable endpoint to assess renal damage.

In the quest for new therapeutic targets to ameliorate CS-induced injury, our attention was drawn to the mitochondrial isoform of the big-conductance Ca^2+^-activated K^+^ (mitoBK) channel, which resides in the inner mitochondrial membrane (for review, see Balderas et al. [17]). Many studies indicate that the endogenous and pharmacological activation of the mitoBK channel protects against cardiac ischemia-reperfusion (IR) injury and other oxidative injuries by reducing mitochondrial ROS, mitochondrial respiratory dysfunction, mitochondrial depolarization, and cell death, ultimately improving cardiac function [18,19,20,21,22,23,24,25,26,27,28]. In this regard, CS-induced renal injury is now thought to be a distinct form of IR injury, which raises the possibility that mitoBK may be a relevant therapeutic target [5]. Thus, we decided to first determine whether renal CS impacts mitoBK channel expression and function and whether the usage of mitoBK activators during CS is protective against CS-induced renal cell injury. 

We investigated the effect of CS + RW on mitoBK channels in vitro using an established rat renal proximal tubular NRK cell model. The NRK cell CS + RW model is intended to recapitulate at the cellular level the intraoperative exposure of donor kidneys to CS-induced ischemia followed by the warm reperfusion step that occurs after transplantation when the donor kidney is reperfused with blood [10,14,16]. Collectively, our findings provide initial evidence that mitoBK channels are expressed in NRK cells and that CS + RW impairs mitoBK channel-mediated K^+^ uptake, which is regarded as a safety valve that prevents Ca^2+^ overload and mitochondrial damage during IR injury [17,29,30]. Importantly, we show that addition of the pharmacological BK channel activator, NS11021, to the CS solution prevents the CS + RW-induced loss of mitoBK channel-mediated K^+^ uptake, and this effect is associated with protection from events associated with mitochondrial injury including mitochondrial respiratory dysfunction, depolarization, and superoxide generation. In addition, NS11021 also partially mitigates CS + RW-induced cell death.

Ultimately, we propose that CS + RW impairs mitoBK function as a novel mechanism contributing to CS-induced mitochondrial injury. These findings raise the possibility that the addition of mitoBK activators to the CS solution can reduce CS-induced renal injury and improve the long-term function of transplanted kidneys. If this therapeutic intervention is shown to be efficacious in vivo, then it could hold immense clinical value due to its simplicity.

## 2. Materials and Methods 

### 2.1. NRK Cell Model of Renal Cold Storage Plus Rewarming 

Normal rat kidney proximal tubular cells (NRK-52E; American Type Culture Collection No. CRL-1571, Manassas, VA, USA) were cultured in 6-well, 100-mm, or 150-mm cell culture dishes (Corning Incorporated, Corning, NY, USA, tissue-culture treated) in a humidified incubator gassed with 5% CO_2_ and 95% air at 37 °C. NRK cells were grown in complete cell culture medium containing Dulbecco’s modified Eagle medium (DMEM; Gibco, Grand Island, NY, USA, #11885), 5% heat-inactivated fetal bovine serum (FBS; Gibco, #10082), and 1% penicillin/streptomycin (Gibco, #15140). Cells were grown to 80% confluency and then randomly assigned to one of the following treatment groups: warm control; cold storage plus rewarming (CS + RW) for 18 h and 2 h, respectively; (CS + RW) + vehicle (DMSO); or CS + RW with the addition of drug(s) to the CS solution.

Control cells remained in DMEM at 37 °C for the same time interval that cells in experimental groups were exposed to CS + RW. CS was initiated by washing cells with cold PBS (Gibco, #10010) and subsequently storing them in cold University of Wisconsin/Viaspan (UW) solution for 18 h at 4 °C. Drug-treated CS + RW groups were stored in UW solution at (4 °C) containing vehicle (0.1% DMSO), NS11021 (1 μM; Sigma, St. Louis, MO, USA, #SML0622), paxilline (5 μM; Cayman Chemicals, Ann Arbor, MI, USA, #11345), or NS11021 (1 μM) + paxilline (5 μM). After 18 h of CS, RW was initiated by washing away the UW solution with cold PBS, replacing the PBS immediately with cold complete cell culture medium, and returning cells to the humidified incubator (5% CO_2_, 37 °C) for 2 h before treated cells and control cells were used in experiments. At first glance, these concentrations of NS11021 (1 μM) and paxilline (5 μM) may seem excessive for an in vitro model. However, our preliminary studies found 1 μM NS11021 to be more therapeutically effective for CS + RW injury than nanomolar concentrations. In addition, higher concentrations of BK modulators may be required to sufficiently activate or inhibit mitoBK channels during CS compared to normothermic conditions due to the inhibitory effect that cold (4 °C) has on drug solubility, organellular drug diffusion, and channel activation, which is discussed further in the section on pharmacological limitations. 

### 2.2. Isolation of NRK Mitochondria

Mitochondrial fractions from NRK cells were prepared by dounce-homogenization followed by differential centrifugation in a mannitol-sucrose buffer (pH 7.4) composed of (in mM): (mannitol 225, sucrose 75, HEPES 10, EGTA 0.1). Briefly, NRK cell homogenates were pelleted twice to remove nuclei, cell debris, and remaining intact cells (750 g, 10 min, 4 °C). Subsequently, the cytosolic and mitochondrial fractions were separated by centrifugation at 10,000× *g* (15 min, 4 °C). The cytosolic fractions were subjected to ultracentrifugation (100,000× *g*, 30 min, 4 °C) to further purify cell debris, flash-frozen in liquid nitrogen and stored at −80 °C until use. In parallel, the mitochondrial pellet obtained from the above protocol was subjected to two more spin-wash steps (10,000× *g*) to further purify the mitochondrial fraction. All steps of the mitochondrial fractionation procedure were performed at 4 °C. The mitochondrial fractions were used either fresh for fluorescence experiments or were flash-frozen in liquid nitrogen and stored at −80 °C until used in Western blots.

### 2.3. Protein Lysates and Immunoblotting

Protein lysates from NRK mitochondrial fractions were prepared by using a radioimmuno-precipitation assay (RIPA) buffer composed of (from Sigma) 1 mM phenylmethylsulfonyl fluoride (PMSF; #P7626), 1 mM 1,4-dithiothreitol (DTT; #D8255), and 1× Halt™ protease and phosphatase inhibitor cocktail (Thermoscientific, Rockford, IL, USA, #78442). Protein concentrations were determined by Coomassie Plus™ protein assay reagent (Thermoscientific, #23236). Mitochondrial fraction-derived protein lysates (20 μg) or cytosolic fraction protein extracts (20 μg) were resolved via SDS-PAGE (200V; 30 min) using precast Bolt™ 8% Bis-Tris Plus gels (Invitrogen, Carlsbad, CA, USA, #NW00080BOX) followed by wet-tank electrophoretic transfer (100V; 2 h) to a 0.2 μm polyvinylidene difluoride (PVDF) membrane (Bio-Rad, Hercules, CA, USA, #1620174). Following transfer, membranes were blocked with 5% non-fat dry milk in TBS-T (0.05% Tween-20) for 1 h at room temperature. Western blotting was performed using antibodies against the following proteins: the pore-forming subunit of the BK channel, BKα (1:1000; Alomone Labs, Jerusalem, Israel, #APC-107), manganese superoxide dismutase, MnSOD (1:1000; EMD Millipore, Burlington, MA, USA, #06–984), proteasome subunit beta type-5, PSMB5 (1:1000; Abcam, Cambridge, UK, #ab3330), and β-actin (1:1000; Sigma, #A5441). MnSOD expression was used as a mitochondrial loading control and marker, and PSMB5 expression was used as a cytosolic marker. β-Actin expression was used as a standard loading control. The probed membranes were washed three times, and immunoreactive proteins were detected using horseradish peroxidase-conjugated secondary antibodies (1:30,000; Seracare KPL, Gaithersburg, MD, USA) and enhanced chemiluminescence (SuperSignal™ West Pico PLUS Chemiluminescent Substrate, Thermoscientific, #34580). Densitometry was calculated using AlphaEase FC software V. 3.1.2 (Alpha Innotech, San Leandro, CA, USA).

### 2.4. Isolation of NRK Cells and Rat Renal Vascular Smooth Muscle Cells 

Patch-clamp experiments used freshly trypsinized NRK cells immediately after exposure to CS + RW, and the results were compared to findings in control NRK cells exposed only to 37 °C culture media. NRK cells were pelleted (600× *g*, 10 min) to remove cell culture media and trypsin, and then resuspended in dissociation solution (DS, described below) before used in patch-clamp experiments. In other experiments, renal vascular smooth muscle cells (VSMCs) were freshly isolated from adult male Sprague Dawley rats using an enzymatic tissue digestion protocol. The animal protocol (#3672) was approved by the Institutional Animal Care and Use Committee (IACUC) at the University of Arkansas for Medical Sciences (UAMS), and all animal experiments were performed in compliance with institutional and National Institutes of Health guidelines. Briefly, renal arteries from both kidneys were quickly dissected from euthanized rats and were cut into 1-mm segments. The arterial segments were gently stirred in a DS composed of (in mM): NaCl 145, KCl 4.0, CaCl_2_ 0.05, MgCl_2_ 1.0, glucose 10, and HEPES 10 (pH adjusted to 7.4 with NaOH) + 0.05% BSA for 10 min at room temperature. The DS was then replaced with new DS containing (in mg/mL): papain 1.23 (Worthington, Lakewood, NJ, USA, #LS003119) and DTT 1.0 (Sigma, #D8255) for 37 min at 37 °C. Finally, renal arteries were incubated in DS containing (in mg/mL): collagenase H 1.77 (Sigma, #C8051), trypsin inhibitor 1.0 (from Soybean; Sigma, #T9003), and elastase 0.5 (Worthington, #LS002292) for 10 min at 37 °C. Finally, the enzymatically digested tissue was briefly incubated in DS containing 0.05% BSA on ice and gently triturated with a fire-polished Pasteur pipette to dislodge renal VSMCs from the arterial tissue. The DS containing freshly isolated renal VSMCs was kept on ice and promptly used for patch-clamp experiments. 

### 2.5. Patch-Clamp Measurement of Whole-Cell BK Channel Current 

To determine whether NRK cells express functional plasmalemmal BK channels in addition to mitoBK channels, patch-clamp protocols designed to detect whole-cell K^+^ current were applied to freshly trypsinized NRK cells pre-exposed either to control (37 °C) or CS + RW conditions. Similar experiments were performed using freshly isolated rat renal VSMCs, which densely express plasma membrane BK channels, and therefore, were used as a positive control for the presence of BK channel-mediated K^+^ current. The whole-cell configuration was obtained by sealing cells with a ~5 MΩ fire-polished borosilicate glass pipette followed by applying suction to the pipette to rupture the membrane patch and obtain the whole-cell patch-clamp mode. Cells were submerged in bath solution composed of (in mM) NaCl 140, KCl 4.0, CaCl_2_ 2.0, MgCl_2_ 1.0, HEPES 10, glucose 10. The pH was adjusted to 7.4 by the addition of NaOH. Cells were dialyzed with pipette solution containing (in mM) glutamate 145, MgCl_2_ 1.0, HEPES 1.0, EGTA 1.0, and Na_2_ATP 1.0. The pH was adjusted to 7.4 by the addition of KOH. The free Ca^2+^ concentration range of the pipette solution was 10–300 nM. The amount of CaCl_2_ required to achieve a specific free Ca^2+^ concentration was calculated using the Maxchelator Ca-Mg-ATP-EGTA calculator v1.0 with constants borrowed from the National Institute of Standards and Technology database 46v8 [31].

Patch-clamp studies used an Olympus IMT-2 inverted microscope (Tokyo, Japan), an L/M-EPC7 amplifier (List Medical, Darmstadt, Germany), and a TL-1 DMA interface (Axon Instruments/Molecular Devices, Sunnyvale, CA, USA). Dialyzed cells were clamped at a holding potential of −70 mV and were progressively depolarized in 10-mV steps to +80 mV. Cells were subjected to the voltage protocol every 2 min to establish initial baseline amplitudes of voltage-activated outward K^+^ current prior to superfusion of cells either with the BK channel activator, 1 μM NS11021 (Sigma, #SML0622), or with the BK channel blocker, 300 nM iberiotoxin (Tocris Bioscience, Bristol, UK, #1086). NS11021 and iberiotoxin were used to delineate which component of the elicited voltage-activated outward K^+^ current was mediated by plasmalemmal BK channels. Data signals were filtered at 1 kHz using a Frequency Devices 902 low-pass filter (Ottawa, IL, USA) before digitization. The Clampex (v. 6) program was used to record traces. Representative traces were prepared for data presentation using Clampfit 10.3.1.5 (Molecular Devices, San Jose, CA, USA) software.

### 2.6. Measurement of mitoBK Channel-Mediated K^+^ Uptake in Isolated NRK Mitochondria

MitoBK channel -mediated K^+^ uptake in isolated NRK mitochondria was measured using a protocol adapted from Aon et al. [32]. Freshly isolated mitochondria from NRK cells were loaded with 20 μM of the intracellular K^+^-sensitive probe, 1,3-benzenedicarboxylic acid, 4,4’-[1,4,10,13-tetraoxa-7,16-diazacyclooctadecane-7,16-diylbis(5-methoxy-6,2-benzofurandiyl)]bis, tetrakis[(acetyloxy)methyl] ester (PBFI-AM; Cayman Chemicals, Ann Arbor, MI, USA, #21602), in a K^+^ -free PBFI buffer (pH, 7.4) composed of 225 mM mannitol and 75 mM sucrose during incubation at room temperature for 20 min in the dark. To improve PBFI-AM aqueous solubility, 0.05% pluronic™ F-127 (Invitrogen, #P6867) was added to the PBFI-AM staining buffer. After staining, the PBFI-staining buffer was removed via centrifugation (14,000× *g*, 5 min), the PBFI-loaded mitochondrial pellet was subjected to another centrifugation-wash step, and then the pellet was re-suspended in K^+^-free PBFI buffer. Mitochondrial K^+^ uptake in the PBFI-loaded mitochondria from NRK cells was then assessed at several time points using a Hitachi F-2500 fluorescence spectrophotometer (Hitachi High-Technologies, Tokyo, Japan). Excitation wavelengths of 340 and 380 nm were used to assess K^+^-binding by the PBFI dye while monitoring emission at 495 nm (ex/em slit lengths of 10/20 nm). Upon binding K^+^, PBFI’s maximal excitation wavelength shifts from 340 to 380 nm. Thus, an increase in the ratio of PBFI fluorescence at 380/340 nm excitation indicates mitochondrial K^+^ uptake. 

Spectrofluorometric measurements of the PBFI-loaded mitochondria were acquired as indicated under the following conditions: (1) K^+^-free baseline, (2) after addition of 10 mM KPO_4_, and (3) after titrations of increasing NS11021 concentrations (100 nM, 500 nM, and 1 μM) to elicit concentration-dependent K^+^ uptake into mitochondria. PBFI fluorescence during each condition was measured every 30 s for 3 min after initiating each condition, and fluorescence generally stabilized within 30–60 s. The data from each experiment presented in Figure 2 were averaged from each reading acquired after PBFI fluorescence stabilized for each condition. To define the component of the NS11021-induced K^+^ uptake mediated by mitoBK channels, K^+^ uptake in PBFI-loaded mitochondria pretreated with 1 μM paxilline (mitoBK blocker) was compared to non-pretreated PBFI-loaded mitochondria for each experimental group. Accordingly, we considered the component of NS11021-elicited K^+^ uptake inhibited by paxilline as “mitoBK channel-mediated K^+^ uptake”. K^+^ efflux was assumed to remain stable throughout the PBFI protocol (note: Δ [K^+^]_mito_ = (K^+^ influx) − (K^+^ efflux)). Ultimately, data were presented (e.g., Figure 2) from studies using 500 nM NS11021, which was the highest concentration of NS11021 at which paxilline fully inhibited NS11021-elicited mitochondrial K^+^ uptake (indicating mitoBK channel -mediated effects) and due to reports that NS11021 concentrations of ≥1 μM can elicit nonspecific effects when applied to isolated mitochondria [32].

### 2.7. High-Resolution Respirometry 

The mitochondrial respiratory complex activity was measured in digitonin-permeabilized NRK cells by high-resolution respirometry (HRR) (Oroboros instruments—Oxygraph-2k, Innsbruck, Austria) using a substrate-inhibitor-titration (SIT) protocol described earlier with minor adaptations for use with NRK cells [11,12,33]. The SIT protocol enables the measurement of oxidative phosphorylation-dependent O_2_ flux (mitochondrial respiration) for each individual complex (Complexes I–IV) of the electron transport chain (ETC).

Briefly, NRK cells were trypsinized after control conditions (37 °C) or CS + RW treatment, washed once with MiRO5 buffer (pH, 7.0) composed of (in mM): K-lactobionate 60, EDTA 0.5, MgCl_2_ 3.0, taurine 20, KH_2_PO_4_ 10, HEPES 20, sucrose 110, and 1 g/L BSA), and subsequently permeabilized with 0.05% digitonin in MiRO5 buffer for 15 min at 4 °C. Subsequently, the permeabilization buffer was removed by centrifugation (600× *g*, 4 °C), the cell pellet was resuspended in O_2_-saturated MiRO5 medium, and 5–10 million cells for each treatment group were collected and added to the oxygraph chamber containing O_2_-saturated MiRO5 medium at 37 °C.

Mitochondrial respiration was initiated by adding 2 mM malate and 10 mM glutamate, and maximal oxidative phosphorylation-dependent respiration was achieved by adding 5 mM ADP. Subsequently, rotenone (0.5 μM) was added to completely inhibit Complex I respiration. Complex II and III respiration was initiated by adding 5 mM succinate followed by 1 mM malonate to inhibit Complex II respiration, and subsequently, 2.5 μM antimycin A to inhibit Complex III respiration. Complex IV respiration was stimulated by adding 50 μM tetramethyl-*p*-phenylenediamine (TMPD; ascorbate-stabilized) followed by inhibition with 250 mM azide. Inhibitor concentrations were selected based on previous experimental determination of the concentrations required to maximally reduce substrate-induced respiration [33]. The DATLAB 4.2 software (Oroboros) was used for data analysis, and mitochondrial respiration for each individual ETC complex was expressed as oxygen flux (pmol/million cells/s).

### 2.8. Detection of Mitochondrial Superoxide Production

MitoSOX Red (Invitrogen, #M36008) was used to detect CS + RW-induced mitochondrial superoxide production in NRK cells. This dihydroethidium dye modified with a cationic mitochondrial-targeting moiety is rapidly taken up by the mitochondria of live cells. Upon oxidation by superoxide, MitoSOX Red subsequently reacts with nucleic acid to produce a bright red fluorescence. Briefly, live NRK cells grown in 6-well dishes on 9 × 22 mm Bellco coverslips (Electron Microscopy Sciences, Hatfield, PA, USA, #7219022) were preloaded with 5 μM MitoSOX Red for 10 min at 37 °C in Hanks’ balanced salt solution (HBSS; Gibco, #14025), washed with HBSS, and then subsequently exposed to CS + RW. Fluorescence spectrophotometry was used to quantify the superoxide-specific MitoSOX (Mito-2-OH-Eth^+^) signal and delineate it from the non-specific MitoSOX (Mito-Eth^+^) signal. A Hitachi F-2500 fluorescent spectrophotometer (Hitachi High-Technologies) equipped with a coverslip holder was used for spectrofluorometric evaluation of mitochondrial superoxide production in MitoSOX-preloaded NRK cells after CS + RW. Excitation/emission wavelengths of 396/580 and 510/580 nm were used to measure the superoxide-specific MitoSOX signal and the non-specific MitoSOX signal, respectively [34]. Excitation/emission slit lengths were 10 and 20 nm, respectively.

### 2.9. Measurement of Mitochondrial Membrane Potential

The relative resting mitochondrial membrane potential of NRK cells and CS + RW-induced mitochondrial depolarization were determined using a lipophilic cationic potentiometric probe 5, 5′, 6, 6′-tetrachloro-1,1′,3,3′-tetraethylbenzimdazol-carbocyanine iodide (JC-1; Invitrogen, #T3168). Green fluorescent JC-1 monomers actively taken up by the hyperpolarized mitochondria aggregate emit red fluorescence. When stained with JC-1, cells with healthy, hyperpolarized mitochondria will display a robust red/orange fluorescence signal, whereas cells with depolarized mitochondria will show a faint, green signal. Briefly, after exposure to CS + RW or control (37 °C) conditions, live NRK cells grown in 6-well dishes on 9 × 22 mm Bellco coverslips were stained with 7.5 μM JC-1 in HBSS for 30 min at 37 °C. Fluorescence spectrophotometry via a Hitachi F-2500 spectrofluorometer equipped with a coverslip holder was used to compare the relative mitochondrial membrane potential between treatment groups. Mitochondrial membrane potential was measured as the ratio of JC-1 aggregate (red; Ex/Em 535/590 nm) to JC-1 monomer (green; Ex/Em 485/530 nm) fluorescence using excitation/emission slit lengths of 10 and 20 nm, respectively.

### 2.10. Measurement of NRK Cell Cytotoxicity

NRK cytotoxicity (cell death) was determined as described earlier using the LDH-Cytotoxicity colorimetric assay kit II (BioVision Research products, Milpitas, CA USA, #K313), which measures lactate dehydrogenase (LDH) release by dying or damaged cells [10]. This assay uses a water-soluble tetrazolium salt (WST) reagent, which reacts with the NADH molecules produced by the LDH enzyme to generate a yellow-colored product (detected at OD450), which quantitatively correlates to the amount of LDH released by damaged cells. OD450 was measured by a SpectraMax M2e microplate reader interfaced with SoftMax Pro 5.4.5 software (Molecular Devices, San Jose, CA, USA). Percent of cytotoxicity was calculated according to the manufacturer’s recommendations, but in brief, it reflects the amount of LDH released into the supernatant media divided by the total LDH present in supernatant media plus intact cells. NRK cell death also was asssessed qualitatively using phase-contrast microscopy (20×) imaging software immediately prior to processing NRK cells for the LDH release assay.

### 2.11. Statistical Analysis

Results are presented as mean ± standard error. Data were analyzed by one-way ANOVA and Tukey’s post-hoc test for multiple group comparisons using GraphPad Prism 8 software (GraphPad Software, San Diego, CA, USA). Differences at the *p* < 0.05 level were considered statistically significant.

## 3. Results

### 3.1. MitoBK Channels Are Expressed in NRK Cells

**Figure 1:**
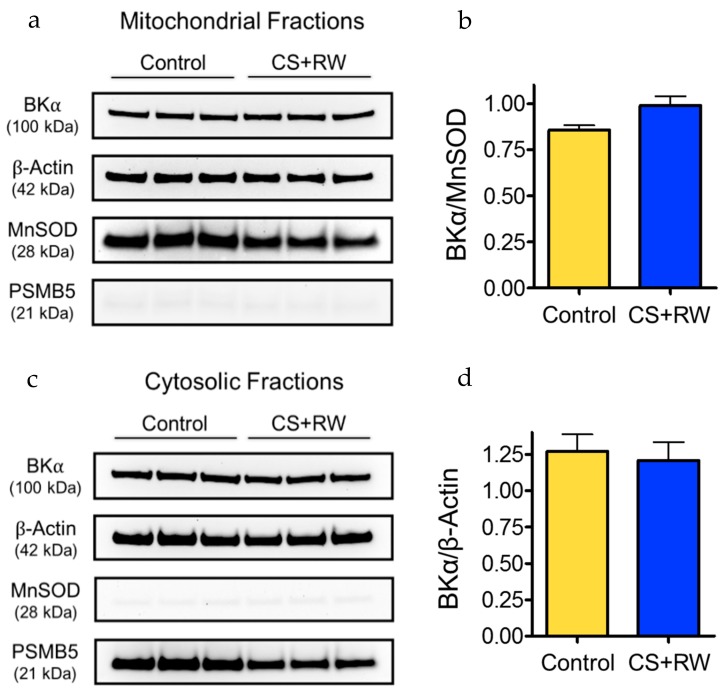
BK channels are detected in mitochondrial fractions from normal rat kidney proximal tubular epithelial (NRK) cells. Western blot shows expression of the pore-forming BKα subunit in mitochondrial fractions (**a**) and cytosolic fractions (**c**) from control NRK cells and after exposure to cold storage and rewarming (CS + RW). Manganese superoxide dismutase (MnSOD) served as a mitochondrial marker and loading control for mitochondrial fractions. Proteasome subunit beta type-5 (PSMB5) was used as a cytosolic marker and β-actin was used as a standard loading control. Representative blots are shown using *n* = 3, where each lane is loaded with 25 μg protein corresponding to a separate experiment. Densitometry analyses for the mitochondrial (**b**) and cytosolic (**d**) fractions are next to corresponding blots; densitometry calculated from two separate blots with a total *n* = 6; no significant differences detected using *p* < 0.05.

We used Western blotting to verify the presence of the BK channel in mitochondria of control NRK cells and to determine whether the expression level of the mitoBK channel is altered in NRK cells exposed to 18 h of CS followed by 2 h of rewarming (CS + RW). The expression of the pore-forming α subunit of the BK channel (BKα) was detected in NRK mitochondrial fraction protein lysates (Figure 1a). BKα expression was similarly detected in NRK cytosolic fractions (Figure 1c). The major mitochondrial antioxidant matrix protein, MnSOD, was used as the mitochondrial loading control, PSMB5 (20S proteasome subunit beta-5) was used as a cytosolic marker, and β-actin was used as a loading control for cytosolic fractions. Selective expression of MnSOD in the mitochondrial fractions and of PSMB5 in the cytosolic fractions confirmed the correct isolation of both subcellular fractions of NRK cells. Expression of β-Actin in the mitochondrial fractions was expected since it serves numerous vital functions within the mitochondrial matrix and thus did not necessarily indicate contamination [35,36]. Densitometry showed that CS + RW did not significantly alter BKα expression in NRK mitochondrial fractions or cytosolic fractions (Figure 1b,d). Overall, these data provide novel evidence that NRK cells contain mitoBK channels. The identity of the BKα subunits detected in NRK cytosolic fractions is unknown but may be attributed to persisting membrane fragments in the cytosol originating from non-mitochondrial organelles and the plasma membrane. 

### 3.2. CS + RW Impairs MitoBK Channel-mediated K^+^ Uptake in NRK Mitochondria, which is Prevented by NS11021 Treatment During CS

Here, we explore the K^+^-conducting function of the mitoBK channel protein in mitochondria isolated from NRK cells for the first time and evaluate the impact of CS+RW on mitoBK channel-mediated K^+^ uptake. Our attempts to directly assess mitoBK currents using patch-clamp methods in NRK cell mitoplasts were unsuccessful. Instead, we used the cell-permeant K^+^-binding fluorescent probe PBFI-AM and BK channel modulators to detect changes in [K^+^]_mito_, thereby providing a surrogate measurement to evaluate K^+^ uptake across the mitochondrial membrane. Our protocol was adapted from Aon et al. who first demonstrated the use of PBFI to measure mitochondrial K^+^ uptake mediated through mitoBK channels [32]. 

**Figure 2:**
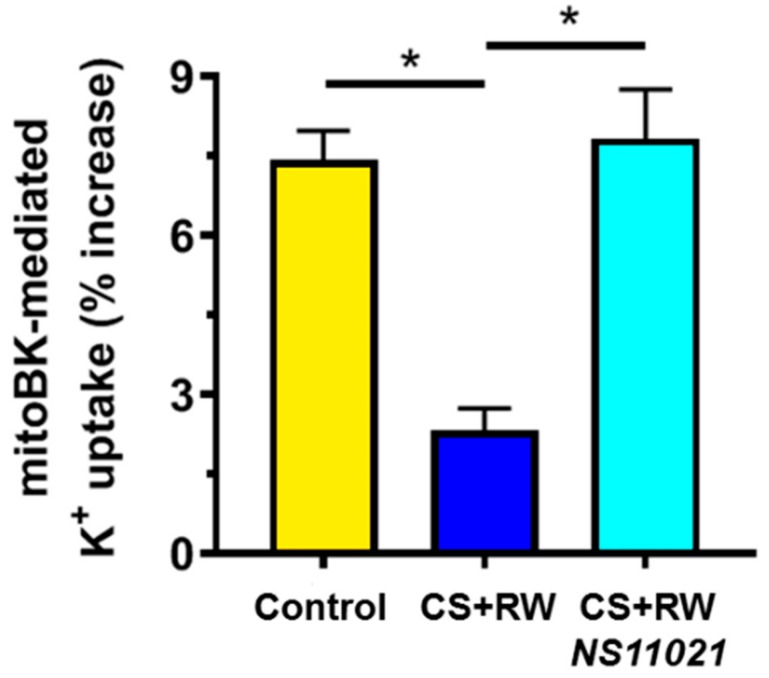
NS11021 protects against CS + RW-induced impairment of mitochondrial big-conductance calcium-activated K^+^ (mitoBK) channel -mediated K^+^ uptake in NRK mitochondria. Fluorescent spectrophotometry was used to measure mitoBK channel -mediated K^+^ uptake (% increase in [K^+^]_mito_) in PBFI-loaded NRK mitochondria isolated from NRK cells that were exposed to the following conditions: control, after CS + RW, or after CS + RW with NS11021 treatment during CS. *n* = 6, * *p* < 0.05.

NRK cells were exposed to CS + RW (18 h + 2 h, respectively), after which fresh mitochondrial fractions were isolated and loaded with PBFI in K^+^-free media. As detailed in the Materials and Methods section, fluorescence spectrophotometry (Ex 340/380 nm, Em 495 nm) was used to measure PBFI fluorescence in PBFI-loaded NRK mitochondria that were exposed to 10 mM K^+^, and subsequently exposed to the BK activator, NS11021, and/or the BK blocker, paxilline. Accordingly, we report the component of NS11021-elicited net K^+^ uptake inhibited by paxilline as “mitoBK channel-mediated K^+^ uptake” (Figure 2). K^+^ efflux was not assessed and assumed to remain stable throughout the PBFI measurements (note, Δ [K^+^]_mito_ = (K^+^ influx) + (K^+^ efflux)). Mitochondria from control NRK cells displayed moderate mitoBK channel-mediated K^+^ uptake, which suggests that NRK cells contain functional mitoBK channels. However, NRK mitochondria from cells that had undergone CS + RW showed impaired mitoBK channel function indicated by the ~69% loss of mitoBK-mediated K^+^ uptake compared to control. Importantly, treatment with NS11021 (1 μM) during CS fully prevented the impairment of mitoBK channel-mediated K^+^ uptake caused by CS + RW, implying the restoration of functional mitoBK channels, which represent a putative mitochondrial-protective mechanism.

### 3.3. NRK Cells do not Express Functional BK Channels in Plasma Membrane

**Figure 3:**
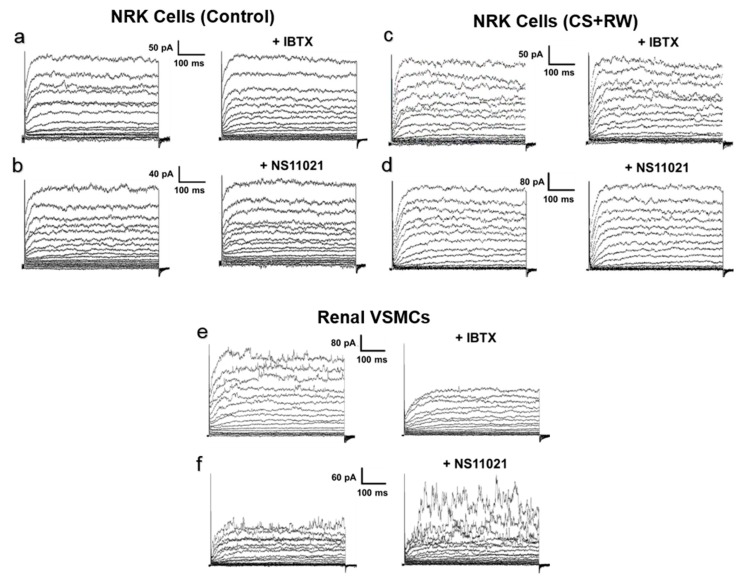
Whole-cell patch-clamp does not detect BK channel-mediated current in plasma membranes of NRK cells. Cells were dialyzed with pipette solution containing 100 nM free calcium, and global K^+^ currents were elicited by 10-mV steps from a holding potential of −70 mV to +80 mV. Representative recordings are shown from (**a**,**b**) control NRK cells and (**c**,**d**) NRK cells subjected to CS + RW. (**a**) and (**c**) show currents before and after 300 nM iberiotoxin (+IBTX), a BK channel blocker. (**b**) and (**d**) show currents before and after 1 μM NS11021, a BK channel opener. NRK cell recordings are representative of *n* = 5–6 for each condition. (**e**,**f**) As a positive control, rat renal vascular smooth muscle cells (VSMCs) were patch-clamped and exposed to NS11021 or iberiotoxin with the same protocol. Renal VSMC recordings are representative of *n* = 3 for each condition.

Since we detected the pore-forming BKα subunit in the cytosolic fraction of NRK cells, which may represent plasma membrane BK channels (Figure 1c), we considered the possibility that NS11021 indirectly affects [K^+^]_mito_ and/or mitochondrial integrity by activating BK channels in the plasma membrane of NRK cells. However, application of the whole-cell patch-clamp technique to isolated NRK cells did not detect BK channel-mediated K^+^ current across plasma membranes of either control NRK cells (Figure 3a,b) or NRK cells subjected to CS + RW (Figure 3c,d). In both NRK cell preparations, the macroscopic K^+^ current elicited by progressive depolarizing voltage steps was insensitive to the selective BK channel blocker, iberiotoxin (IBTX, 300 nM). IBTX was used for whole-cell patch clamp experiments since it is the most effective known blocker of plasmalemmal BK channels. IBTX was not used in other experiments due to its membrane-impermeability; instead the membrane-permeable BK blocker, paxilline, was used. Additionally, NS11021 (1 μM) did not enhance K^+^ current amplitudes in NRK cells, further suggesting the absence of functional BK channels on the NRK cell surface. Rat renal vascular smooth muscle cells (VSMCs), a cell preparation known to densely express functional BK channels in the plasma membrane, were similarly patch-clamped as a positive control (Figure 3e,f). We readily detected K^+^ currents that were inhibited by iberiotoxin and enhanced by NS11021, which verified that our technique could detect plasmalemmal BK channel-mediated K^+^ currents. Ultimately, the data indicated that NRK cells do not contain functional BK channels in the plasma membrane, which makes the NRK CS + RW model conducive for pharmacologically evaluating mitoBK as a therapeutic target.

### 3.4. NS11021 Attenuates CS + RW-Induced Mitochondrial Respiratory Dysfunction

**Figure 4:**
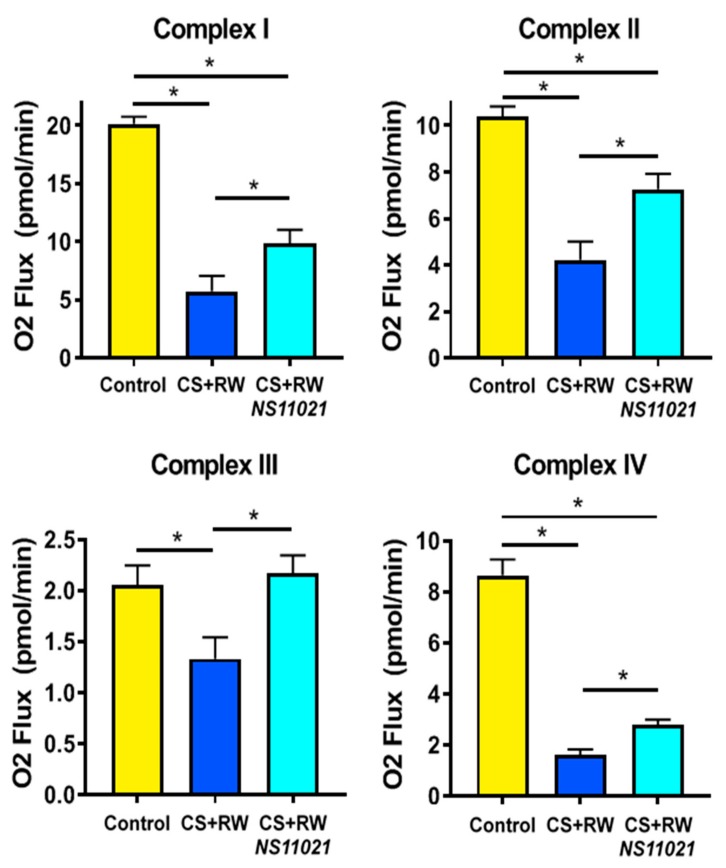
The BK channel activator, NS11021, partially prevents CS + RW-induced mitochondrial respiratory dysfunction at Complexes I–IV. High resolution respirometry (HRR) was used to measure mitochondrial respiration (O_2_ flux) at electron transport chain Complexes I–IV in NRK cells. Data are shown for the following conditions: control, after CS + RW, and after CS + RW treated with NS11021 (1 μM) during CS. *n* = 6, * *p* < 0.05.

High resolution respirometry (HRR) was used to evaluate mitochondrial respiration in live digitonin-permeabilized NRK cells to determine whether NS11021-elicited activation of mitoBK channels during CS confers protection against CS-induced mitochondrial respiratory dysfunction. We previously reported that CS + RW impairs mitochondrial respiration in NRK cells; however, this method utilized isolated mitochondria in which Complex I–IV respiration was assessed spectrophotometrically [10]. High resolution respirometry of live cells is particularly advantageous since it does not introduce respirometric artifacts known to occur during mitochondrial isolation [37,38]. To our knowledge, this is the first study to assess the impact of CS + RW on mitochondrial respiration at Complexes I–IV. Compared to control cells maintained at 37 °C, NRK cells subjected to CS + RW exhibited severe impairment of mitochondrial respiration at Complexes I, II, III, and IV, as evident by the 72, 60, 36, and 82% reductions in O_2_ flux, respectively (Figure 4). The addition of NS11021 (1 μM) to the CS solution was partially protective against CS + RW-induced respiratory dysfunction in NRK cells at Complexes I, II, and IV and conferred full protection at Complex III.

### 3.5. NS11021 Mitigates CS + RW-Induced Mitochondrial Superoxide Production

**Figure 5:**
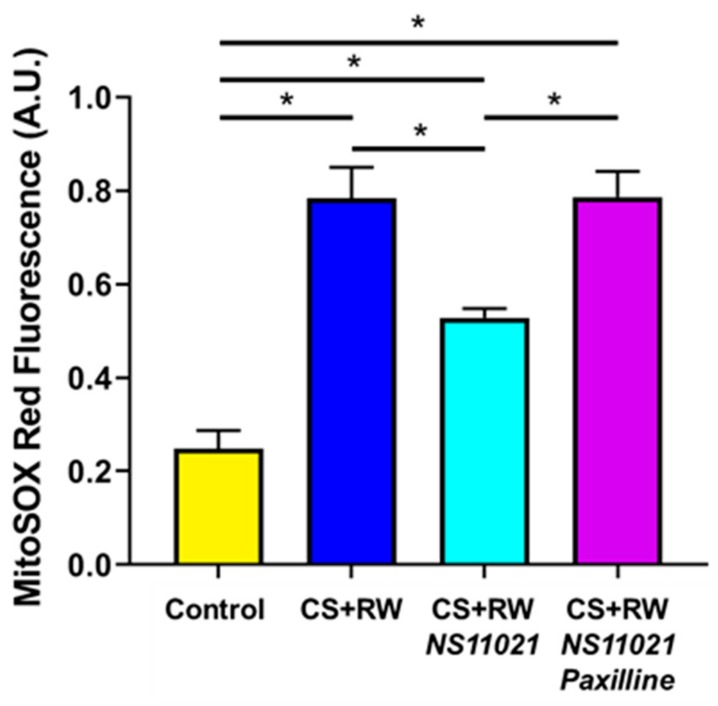
NS11021 protects against CS + RW-induced mitochondrial superoxide production. The mitochondrial superoxide-specific MitoSOX signal was quantified via fluorescence spectrophotometry using excitation/emission wavelengths of 396/580 nm. Data are shown for NRK cells in the following conditions: control, after CS + RW, after CS + RW treated with 1 μM NS11021 during CS, and after CS+RW treated with 1 μM NS11021 and 5 μM paxilline during CS. *n* = 7, * *p* < 0.05.

We reported earlier that CS + RW increases mitochondrial superoxide production in NRK cells, which is a key indicator of mitochondrial injury and cellular stress [10]. Here, we similarly used the mitochondrial superoxide-specific fluorescent probe, MitoSOX Red, to evaluate whether mitoBK channel activation via the addition of NS11021 to the CS solution alters mitochondrial superoxide generation during CS + RW in NRK cells. As detailed in Materials and Methods, fluorescence spectrophotometry with excitation/emission of 396/580 nm was used to detect the mitochondrial superoxide-specific MitoSOX (Mito-2-OH-Eth^+^) signal and delineate it from the non-specific (Mito-Eth^+^) signal [34]. Compared to control NRK cells, NRK cells exposed to CS + RW exhibited a roughly 3-fold increase in MitoSOX fluorescence indicative of elevated mitochondrial superoxide production (Figure 5). Addition of NS11021 (1 μM) during CS mitigated CS + RW-induced mitochondrial-superoxide as indicated by a ~50% reduction in CS + RW-induced MitoSOX fluorescence. The protective effect of NS11021 was fully antagonized when the BK channel blocker, paxilline (5 μM), was combined with NS11021 in the CS solution. These data indicate that the ability of NS11021 to mitigate CS + RW-induced mitochondrial superoxide is dependent on its BK channel -activating properties.

### 3.6. NS11021 Mitigates CS + RW-Induced Mitochondrial Depolarization

**Figure 6:**
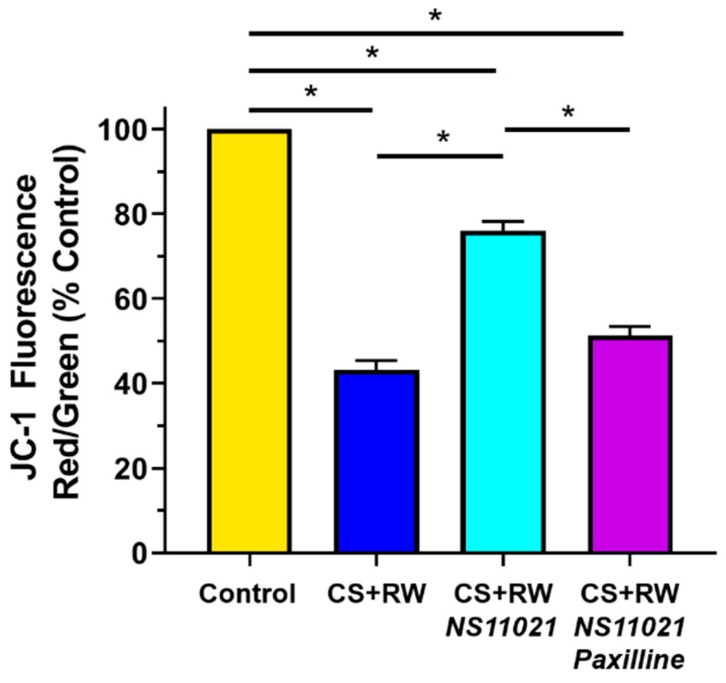
NS11021 protects against CS + RW-induced mitochondrial depolarization. Relative mitochondrial membrane potential was quantified using fluorescence spectrophotometry to detect the ratio of JC-1 aggregates (red; ex/em 535/590 nm) to monomers (green; ex/em 485/530 nm). Data are shown for NRK cells in the following conditions: control, after CS + RW, after CS + RW treated with 1 μM NS11021 during CS, and after CS + RW treated with 1 μM NS11021 and 5 μM paxilline during CS. *n* = 6, * *p* < 0.05.

Next we used the mitochondrial-targeted potentiometric fluorescent probe JC-1 to evaluate the impact of CS + RW on NRK mitochondrial membrane potential and determine whether NS11021 treatment during CS could protect against CS + RW-induced mitochondrial depolarization. Mitochondrial membrane potential was calculated via fluorescence spectrophotometry as the ratio of JC-1 aggregates (red; 535/590 nm) to JC-1 monomers (green; 485/530 nm) emissions as detailed in the Materials and Methods section. As expected, control NRK cells exhibited a hyperpolarized mitochondrial membrane potential (Figure 6). In contrast, NRK cells subjected to CS + RW exhibited profound mitochondrial depolarization, with a ~60% loss of relative mitochondrial membrane potential. Treatment with NS11021 (1 μM) during CS partially attenuated CS + RW-induced mitochondrial depolarization, providing ~ 65% protection. The addition of paxilline (5 μM) to the CS solution fully antagonized the protection offered by NS11021, indicating that the ability of NS11021 to mitigate CS + RW-induced mitochondrial depolarization is dependent on its BK channel -activating properties.

### 3.7. NS11021 Attenuates CS + RW-Induced Cell Death

**Figure 7:**
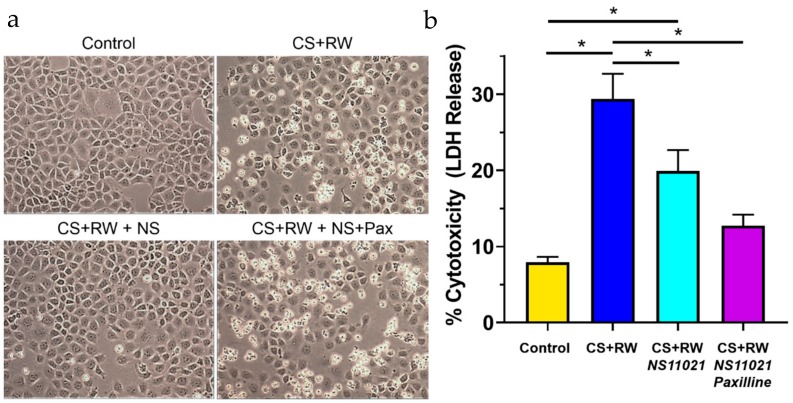
NS11021 protects NRK cells against CS + RW-induced cytotoxicity. (**a**) Microscopic evaluation for qualitative assessment of CS + RW-induced cell death. Representative images of NRK cells were acquired prior to performing an LDH release assay after cells were subjected to one of the following conditions: control, after CS + RW, after CS + RW with 1 μM NS11021 added to the CS solution, and after CS + RW with 1 μM NS11021 and 5 μM paxilline added to the CS solution; *n* = 6. (**b**) Cytotoxicity was assessed by a colorimetric LDH release assay. Data are shown for the four experimental conditions corresponding to (**a**). *n* = 6, * *p* < 0.05.

CS + RW-induced cytotoxicity was assessed qualitatively by microscopic evaluation of NRK cell morphology and quantitatively by a commercial colorimetric kit (BioVision), which measures lactate dehydrogenase (LDH) release from damaged/dying cells. The proportion of LDH released into the supernatant media compared to total cellular LDH is indicative of percent cytotoxicity or cell death. Qualitatively, CS + RW resulted in substantial NRK cell death (Figure 7a). The addition of 1 μM NS11021 to the CS solution partially prevented the loss of NRK cells after CS + RW, a protective effect abolished by the BK channel blocker, paxilline. An LDH release assay was performed immediately after image acquisition to quantify CS + RW-induced cytotoxicity (Figure 7b). This assay revealed that CS + RW resulted in a 3.7-fold increase in LDH release compared to control NRK cells maintained at 37 °C. The addition of 1 μM NS11021 to the CS solution reduced CS + RW-induced LDH release by 40%. Unexpectedly, 5 µM paxilline did not reverse the protection conferred by NS11021 against CS + RW–induced LDH release, a puzzling discrepancy which is addressed in the Discussion section.

## 4. Discussion

The present study has four principal new findings. We provide the first evidence, to our knowledge, that functional mitoBK channels are expressed in mitochondria of NRK cells. Second, we demonstrate that CS + RW impairs mitoBK channel–mediated K^+^ uptake in NRK cells, which is among the first reported findings regarding the impact of clinically-relevant disease (CS-induced renal injury) on mitoBK channel function. Third, our findings indicate that addition of the BK channel opener, NS11021, to CS solution can restore mitoBK channel–mediated K^+^ uptake into mitochondria of NRK cells. Fourth, prophylactic therapy using NS11021 in CS solution partially protects NRK cells against CS + RW-induced cell death and mitochondrial injury by preserving mitochondrial respiration and membrane potential and attenuating mitochondrial superoxide generation. Collectively, our findings raise the possibility that the addition of small molecule therapeutics to the CS solution to restore mitoBK channel activity in renal tubular cells subjected to CS + RW may alleviate renal cell CS injury.

### 4.1. NRK Cells Express MitoBK Channels

Our initial studies confirmed for the first time that mitoBK channels were expressed in NRK cells. In these studies, mitochondrial fractions from NRK cells revealed a 100-kDa immunoreactive band on Western blots corresponding to the pore-forming BKα subunit. Other laboratories have reported similar BKα immunoreactive signals between 100 and 150 kDa (depending on the primary antibody used) in cardiomyocyte mitochondria [19,21]. For mitoBK channels in cardiomyocytes, mitochondrial localization was found to require a C-terminal splice variant referred to as the DEC insert [21]. However, the mechanisms involved in mitochondrial-targeting of mitoBK channels may vary between different cell types [17]. For all BK channel isoforms, whether on the plasma membrane or inner mitochondrial membrane, it is assumed that four α subunits are required to form the pore structure [17,39]. However, important physiological properties of BK channels including their voltage and Ca^2+^-sensitivity depend on one-to-one co-assembly of the four BKα subunits with four regulatory BKβ subunits, which can include one of four isoforms (BKβ1, β2, β3, β4). Considering that different BKβ isoforms can profoundly impact the BK channel phenotype, including cellular localization and electrophysiological properties such as voltage sensitivity, Ca^2+^ sensitivity, and inactivation kinetics [40], an important next step would be to identify the precise BKβ isoform(s) that regulate mitoBK channels in renal tubular cells. Recently, a study utilizing BKβ1-KO mice reported that the BKβ1 isoform regulates mitoBK channels and is required for its mitochondrial localization and activation in cardiomyocytes [41]. However, studies have called into question the integrity of BKβ isoform-specific commercial antibodies, which complicates the endeavor of assessing mitoBKβ isoforms via Western blot [42]. 

Notably, we also observed that the expression level of BKα subunits in mitochondrial fractions from NRK cells did not differ between control NRK cells and NRK cells exposed to CS + RW. Considering that we recently reported that CS injury disrupts expression of certain mitochondrial proteins, it seemed reasonable that mitoBKα expression could be downregulated by CS [11]. Indeed, the expression of ion channels and other proteins can be rapidly regulated by temperature, and hypothermia can impact ion channel expression and activation [43,44,45]. However, in the present study, we found no evidence to suggest that CS + RW altered the expression of mitoBK channels. Future studies to show the expression of mitoBKα protein in isolated inner mitochondrial membranes and the direct recording of BK channel–mediated currents from mitoplast patches would be important next steps to corroborate the expression of functional mitoBK channels in NRK cells. 

### 4.2. CS + RW Impairs MitoBK Channel–Mediated K^+^ Uptake

In our study, we used the K^+^-binding fluorescent dye, PBFI-AM, to indirectly measure mitoBK-mediated K^+^ uptake in isolated NRK mitochondria. Our protocol was adapted from Aon et al. who assessed mitoBK channel -mediated K^+^ uptake in isolated cardiac mitochondria using NS11021 and paxilline to activate and inhibit the channel, respectively [32]. The paxilline-sensitive component of NS11021-induced changes in [K^+^]_mito_ was attributed to mitoBK channel-mediated K^+^ uptake across the mitochondrial inner membrane. Using PBFI, we observed that CS + RW markedly impairs mitoBK-mediated K^+^ uptake in mitochondria of NRK cells; BKα expression, however, was unaltered by CS + RW. Collectively, these findings infer that CS + RW inactivates functional mitoBK channels. This finding is particularly exciting since it is among the first to report the impact of clinically-relevant disease, namely CS-induced renal injury, on mitoBK channel function. 

Our studies did not address which events during CS + RW inactivate mitoBK channels in NRK cells, but presumably, it involves a stable channel modification since we observed a loss of mitoBK channel -mediated K^+^ uptake after mitochondria were isolated from NRK cells subjected to CS + RW. One possibility is that mitochondrial ROS, which are known to be generated during CS + RW, inactivate mitoBK channels [10,14,46]. The pore-forming BKα protein has numerous cysteine-rich functional domains susceptible to the formation of ROS adducts, which are associated with channel inactivation [46,47,48]. Stressors may also induce post-translational modification (PTM) of BKα subunits by phosphorylation or palmitoylation of amino acid residues in critical functional domains, thereby altering BK channel activity [49]. Additionally, multiple intracellular kinases can modify BK channel activity through post-translational modifications or secondary messengers [50,51,52]. Finally, the hypoxia-sensitive stress-axis-regulated-exon (STREX) splice variant of BKα, which was recently detected in cardiac mitoBK channels, has been implicated in regulating the cytoprotective activation of mitoBK channels during ischemic injury [21,53]. However, the contribution, if any, of these PTMs and signaling pathways to the loss of mitoBK channel-mediated K^+^ uptake in renal cells after CS + RW remains unresolved and should be investigated in future experiments. 

Most studies of mitoBK channels have been limited to normal (non-diseased) brain and cardiac preparations, and have defined the mitoBK channel’s biophysical properties by patch-clamping the mitoplasts composed of inner mitochondrial membrane [17,23,54,55,56,57,58,59,60,61,62,63,64]. Patch-clamping studies of mitoBK channels could illuminate how CS + RW impairs mitoBK channel function, for example, by detailing whether a lower probability of channel opening is due to a loss of voltage or calcium sensitivity. However, we were unable to use patch-clamp methods to record mitoBK channel currents from mitoplasts freshly isolated from NRK cells, although we have extensively patch-clamped other cell preparations. The stability of mitoplasts was short-lived, particularly mitoplasts from NRK cells exposed to CS + RW, perhaps due to the profoundly deleterious effect that CS has on mitochondrial structural integrity [11]. In this regard, few studies—if any—have patch-clamped mitoBK channels on mitoplasts isolated from diseased or severely injured cells or tissues. While electrophysiological studies of mitoBK channels are undoubtedly valuable, they cannot assess the impact of mitoBK channel-mediated current on [K^+^]_mito_, which is an advantage of K^+^ sensitive dyes in addition to their ease of use in disease models. Regardless, future patch-clamp studies can help to shed light on mechanisms by which CS + RW alters the electrophysiological properties of mitoBK channels to compromise mitochondrial K^+^ uptake. 

### 4.3. NS11021 Restores MitoBK Channel-Mediated K^+^ Uptake

A central finding of our study is that mitoBK channel-mediated K^+^ uptake is fully restored in mitochondria of NRK cells subjected to CS + RW by simply adding NS11021 to the CS solution. This finding also supports the concept that CS + RW inactivates rather than results in a loss of mitoBK channels. According to the equation I = n·i·p; where I = K^+^ influx, n = number of channels, i = single-channel current amplitude, and p = open-state probability, the loss of mitochondrial K^+^ uptake caused by CS+RW is most readily attributed to a reduced open-state probability (p) of mitoBK channels. Indeed, our Western blots indicated that CS + RW did not alter BKα expression (n), and current amplitude (i) generally is regarded as the distinctive fingerprint of a channel which does not change. Interestingly, some reports have suggested that mitoBK channels play a protective role when endogenously activated in ex vivo and in vivo mouse models of cardiac IR injury [19,20]. This finding raises the possibility that mitoBK channels in renal tubular cells also are activated during CS + RW as a native protective mechanism, but this response is overwhelmed by the pathogenic processes associated with CS injury. However, the addition of NS11021 to the CS solution further may capitalize on this endogenous protective mechanism to restore mitoBK channel function, an event that our study and other reports link to mitochondrial protection.

It is important to note a key assumption made regarding our PBFI experiments and the determination of mitoBK channel -mediated K^+^ uptake. The PBFI dye measures [K^+^]_mito_, which we use to detect changes in net K^+^ uptake between experimental conditions. By definition, Δ [K^+^]_mito_ = (K^+^ influx) - (K^+^ efflux). Using the BK channel activator, NS11021, and blocker, paxilline, our experimental protocol delineates the portion of net K^+^ uptake which results from K^+^ influx mediated specifically by mitoBK channel opening (which we report as mitoBK-mediated K^+^ uptake). However, this approach assumes that K^+^ efflux mechanisms remain stable during the PBFI experimental protocol. In the current study, we did not assess whether CS differentially impacts mitochondrial K^+^ efflux mechanisms, so it was assumed to remain stable between experimental conditions. To minimize the possibility of altering K^+^ efflux through off-target drug effects, we used low concentrations of NS11021 (500 nM) and paxilline (1 μM) during the PBFI protocol to measure mitoBK channel-mediated K^+^ uptake; these concentrations are reported to be specific for BK channels and have not been shown to directly act on other K^+^ channels or K^+^ transporters involved in K^+^ efflux mechanisms [31,65,66].

### 4.4. NS11021 Protects NRK Cells Against CS + RW-Induced Mitochondrial Injury and Cell Death

Importantly, we observed that addition of NS11021 to the CS solution to pharmacologically activate mitoBK channels protected mitochondria of NRK cells against CS + RW-induced mitochondrial respiratory dysfunction, depolarization, and superoxide production. The concurrent addition of paxilline, a membrane-permeable BK channel inhibitor, to the CS solution antagonized the mitochondrial-protective effects of NS11021. Thus, the therapeutic benefit conferred by NS11021 to NRK cells challenged by CS + RW relies on its ability to activate mitoBK channels. This finding is consistent with other studies, including experiments by Bentzen et al., which showed that the cardioprotective effects of NS11021 in an isolated-perfused rat heart model of IR were mitoBK channel-dependent and antagonized by paxilline [18]. NS11021 also protected cardiomyocytes from simulated IR injury induced by chemical hypoxia by reducing ROS production and mitigating cell death in a paxilline-antagonized manner [22]. Interestingly, at nanomolar concentrations regarded as selective for BK channels, NS11021 improved mitochondrial respiratory efficiency in isolated heart mitochondria under basal, non-diseased conditions [32]. Notably, the current study is the first to demonstrate using a cell model of disease (NRK cells exposed to CS + RW) that NS11021 has distinct mitochondrial-protective effects that are mitoBK channel -dependent and paxilline-antagonized. Our findings particularly serve to draw attention to mitoBK channel openers as potentially effective medications to protect renal cells against CS injury as a strategy to improve the long-term function of renal transplants.

NS11012 also mitigated CS + RW-induced cell death as indicated qualitatively by microscopic evaluation and quantitatively by LDH release, which is consistent with other studies showing that NS11021 has cytoprotective properties during IR-injuries [18,22]. Microscopic evaluation of NRK cell morphology indicates that paxilline clearly reversed the cytoprotective effect of NS11021 against CS + RW-induced cell death, suggesting a BK channel -mediated effect. Unexpectedly, paxilline did not reverse the mitigating effect of NS11021 against CS + RW-induced LDH release, which suggests that BK channels are not solely responsible for the NS11021-mediated protection from CS + RW-induced cytotoxicity. This discrepancy is difficult to reconcile; however, it could be related to the fact that this LDH release assay primarily measures necrotic (not apoptotic cell death), and thus it is possible that paxilline’s off-target effects shifted cell death to a more apoptotic pathway. Regardless, adding NS11021 to the CS solution provided significant protection against CS + RW-induced cell death, and further characterization is needed to ascertain whether this effect is mediated through BK channels.

Although our findings clearly demonstrate that adding NS11021 to CS solution offers protection from CS + RW-induced injury to NRK mitochondria, a finding that we attribute to the activation of mitoBK channels, it should be noted that we also detected the pore-forming BKα protein in NRK cell cytosolic fractions, which may contain membrane fragments from intracellular organelles or plasma membrane. Many studies have indicated that NS11021 also activates plasmalemmal BK channels, so we were concerned that the therapeutic benefit of adding NS11021 to CS solution might at least partially rely on the activation of BK channels in the plasma membrane or other non-mitochondrial organelles [65,67,68]. However, to our surprise, our patch-clamp recordings of voltage-gated macroscopic K^+^ currents in NRK cells did not detect iberiotoxin-sensitive current attributable to cell-surface BK channels. Additionally, NS11021 did not increase plasmalemmal K^+^ current either in NRK cells maintained at 37 °C or those subjected to CS + RW. In contrast, a component of IBTX-sensitive K^+^ current was prominent in rat renal VSMCs, a positive-control preparation known to densely express plasmalemmal BK channels, and NS11021 markedly increased K^+^ current in these cells. Thus, the NRK cell model that lacks potentially-confounding plasmalemmal BK channels may be particularly useful for evaluating mitoBK channels as a therapeutic target. Interestingly, cardiac myocytes also do not express plasmalemmal BK channels, and NS11021 shows therapeutic efficacy against IR injury in these cells and in ex vivo models of cardiac IR [18,21,22]. Nevertheless, our studies did not rule out the possibility that NS11021 activates BK channels at other non-mitochondrial organelles considering that cross-talk between intracellular organelles is well recognized [69,70,71,72,73,74]. Indeed, BK channels have been discovered in many cellular organelles as is nicely reviewed by Sing et al. [75]. Of these structures, it is likely that membranes associated with the endoplasmic reticulum, golgi, lysosomes, and/or peroxisomes, which are low-density organelles, may have remained in the cytosolic fraction during our fractionation process to provide BKα immunoreactivity from other intracellular BK channel isoforms.

### 4.5. Pharmacological Limitations

Our study has several limitations related to its pharmacological approach, which should be acknowledged. First, we chose NS11021 to pharmacologically activate BK channels in our study because it is more potent and selective than earlier prototype molecules in this drug family [65]. Preliminary studies using a range of NS11021 concentrations (100, 300, 500 nM, and 1 μM) justified the 1 μM concentration to optimally protect against CS + RW-induced mitochondrial injury. Although 1 μM NS11021 and 5 μM paxilline appear to be relatively high concentrations to use in vitro, we considered that adding a hydrophobic drug (NS11021) to hyperosmotic CS solution at 4 °C may result in limited solubility and diffusion compared to solutions maintained at 37 °C, effectively reducing drug availability for channel binding. Additionally, compared to plasmalemmal BK channels, it is likely that less drug will reach mitoBK channels during CS using our NRK cell model. Regardless, 1 μM NS11021 is still within the range of concentrations reported to be highly specific for BK channels compared to other ion channels, although off-target effects unrelated to ion channels cannot be ruled out [32,65]. In contrast, most studies pharmacologically assessing mitoBK channels as therapeutic targets to protect against cardiac IR injury used an earlier BK channel activator, NS1619 [17,21,23,24,27,28,29]. In these studies, which are comprehensively reviewed by Bentzen et al. and Balderas et al., NS1619 offered partial protection against IR injury in numerous cell and animal models, ostensibly by activation of mitoBK channels [17,29]. However, NS1619 is now known to exhibit considerable off-target effects including the inhibition of voltage-gated Ca^2+^, K^+^, and Na^+^ channels and Ca^2+^-gated Cl^−^ channels [29]. Furthermore, a study using isolated mitochondria from cardiomyocytes found NS1619 to have extensive mitochondrial effects independent of mitoBK channel opening, including respiratory inhibition, uncoupling, and mitochondrial swelling due to non-selective permeabilization of the inner mitochondrial membrane to ions [76]. Most of these off-target effects of NS1619 were observed at moderate concentrations (5–50 μM) commonly used for therapeutic activation of BK channels [17,29]. A second limitation is that although paxilline is regarded as a specific BK channel blocker, it also can inhibit Ca^2+^ uptake by sarco/endoplasmic reticulum Ca^2+^-ATPase (SERCA) at concentrations ≥5 μM, thereby potentially modifying intracellular Ca^2+^ signaling, a pathway already disrupted in IR injury as well as CS injury [77,78,79,80]. While most studies have used paxilline concentrations between 1 and 5 μM to prevent pharmacological activation of mitoBK channels by NS11021, some studies have used higher concentrations of 10–50 μM for this purpose [18,22,24,27,28,62,63,64,81,82,83,84,85,86]. Considering that few putatively selective, cell-permeable BK channel blockers are commercially available, we chose to use the highest concentration (5 μM) of paxilline reported to effectively prevent pharmacological activation of mitoBK channels while avoiding higher concentrations associated with off-target effects [66,77]. Third, it should be acknowledged that the protection against CS + RW–induced mitochondrial injury of NRK cells conferred by NS11021 in our study may not extend to transplanted kidneys preserved in CS solution containing mitoBK channel openers such as NS11021. A number of factors, including the half-life of the therapeutic molecule in CS solution, distance of drug diffusion into renal tissue, and drug effects on non-tubular cells, including the renal vasculature, will impact therapeutic potential, and ultimately, renal transplant function. Alternatively, a major advantage of limiting mitoBK channel activators to the CS solution rather than systemically administration to a patient is that it averts the systemic side effects reported for these drugs, such as hypotension, while potentially improving renal transplant function.

## 5. Conclusions

Our findings raise the possibility that including NS11021 or other pharmacological activators of mitoBK channels in CS solution used for the preservation of donor kidneys may alleviate CS injury to renal tubular cells. Multiple measures of mitochondrial integrity including mitochondrial respiratory function and mitochondrial membrane potential were protected in NRK cells by adding NS11021 to the CS solution prior to cell rewarming, a process associated with ischemia-reperfusion injury. Additionally, mitochondrial superoxide production and cell death were mitigated by NS11021 in our NRK cell model of CS + RW-induced injury. These findings emphasize the importance of future cell-based analyses to define the mechanisms by which CS + RW inhibits mitoBK channels and preclinical studies using rat models of renal transplantation to evaluate whether adding mitoBK channel activators to CS solution can improve the long-term function of transplanted kidneys.
